# A map of human circular RNAs in clinically relevant tissues

**DOI:** 10.1007/s00109-017-1582-9

**Published:** 2017-08-25

**Authors:** Philipp G. Maass, Petar Glažar, Sebastian Memczak, Gunnar Dittmar, Irene Hollfinger, Luisa Schreyer, Aisha V. Sauer, Okan Toka, Alessandro Aiuti, Friedrich C. Luft, Nikolaus Rajewsky

**Affiliations:** 1 Experimental and Clinical Research Center (ECRC), a joint cooperation between the Charité Medical Faculty and the Max Delbrück Center for Molecular Medicine (MDC), Lindenberger Weg 80, 13125 Berlin, Germany; 2 Max Delbrück Center for Molecular Medicine (MDC), Robert-Rössle-Strasse 10, 13125 Berlin, Germany; 3 Department of Stem Cell and Regenerative Biology, Harvard University, 7 Divinity Ave, Cambridge, MA 02138 USA; 4 Scientific Institute HS Raffaele, San Raffaele Telethon Institute for Gene Therapy (SR-Tiget), 20132 Milan, Italy; 5 Department of Pediatric Cardiology, Children’s Hospital, Friedrich-Alexander University Erlangen, Loschge Strasse 15, 91054 Erlangen, Germany; 6 The German Registry for Congenital Heart Defects, Augustenburger Platz 1, 13353 Berlin, Germany; 7 Vita Salute San Raffaele University, Milan, Italy; 8 Department of Medicine, Division of Clinical Pharmacology, Vanderbilt University School of Medicine, Nashville, TN 37235 USA

**Keywords:** Circular RNAs, circRNA catalog, Potential biomarker, Human cell types

## Abstract

**Abstract:**

Cellular circular RNAs (circRNAs) are generated by head-to-tail splicing and are present in all multicellular organisms studied so far. Recently, circRNAs have emerged as a large class of RNA which can function as post-transcriptional regulators. It has also been shown that many circRNAs are tissue- and stage-specifically expressed. Moreover, the unusual stability and expression specificity make circRNAs important candidates for clinical biomarker research. Here, we present a circRNA expression resource of 20 human tissues highly relevant to disease-related research: vascular smooth muscle cells (VSMCs), human umbilical vein cells (HUVECs), artery endothelial cells (HUAECs), atrium, vena cava, neutrophils, platelets, cerebral cortex, placenta, and samples from mesenchymal stem cell differentiation. In eight different samples from a single donor, we found highly tissue-specific circRNA expression. Circular-to-linear RNA ratios revealed that many circRNAs were expressed higher than their linear host transcripts. Among the 71 validated circRNAs, we noticed potential biomarkers. In adenosine deaminase-deficient, severe combined immunodeficiency (ADA-SCID) patients and in Wiskott-Aldrich-Syndrome (WAS) patients’ samples, we found evidence for differential circRNA expression of genes that are involved in the molecular pathogenesis of both phenotypes. Our findings underscore the need to assess circRNAs in mechanisms of human disease.

**Key messages:**

- circRNA resource catalog of 20 clinically relevant tissues.
- circRNA expression is highly tissue-specific.
- circRNA transcripts are often more abundant than their linear host RNAs.
- circRNAs can be differentially expressed in disease-associated genes.

**Electronic supplementary material:**

The online version of this article (10.1007/s00109-017-1582-9) contains supplementary material, which is available to authorized users.

## Introduction

Cellular circular RNAs (circRNAs) represent a class of single-stranded, unusually stable RNAs originating from 5′-to-3′ transcription of coding gene exons or long non-coding RNAs (lncRNAs) that produce covalently closed head-to-tail (or back-spliced) circularized transcripts [1–4]. Many circRNAs are tissue- and developmental stage-specifically expressed [2, 4]. The circRNA co-transcriptional splicing can compete with linear splicing events and can depend on the binding of the RNA-binding proteins, MBNL1 or QKI, in intronic sequences [5, 6]. Intronic complementary sequences, inter alia repetitive elements, and the RNA-editing protein, ADAR1, were linked to the circularization of exons [7, 8]. Earlier, the circRNAs CDR1as (ciRS-7) and circSRY were shown to exhibit important functions in sponging miRNAs and thereby functioning as post-transcriptional regulators [2, 9, 10]. circRNAs are resistant to the exonuclease RNase R that solely digests linear transcripts. This feature can be used to validate circRNA candidates by comparing their abundance in the RNase R-treated and untreated samples [11]. circRNAs found in clinical specimens, like blood, reveal that these abundant transcripts could serve as biomarkers [12]. Here, we present a circRNA resource catalog to supplement existing databases with new circRNA transcripts in human cell types. By identifying and validating selected circRNAs in these human tissues relevant to clinical research, we provide multiple examples of abundant and highly tissue-specific circRNA expression in host genes that have been associated with pathogenesis of human disease.

## Methods

### Human material

After approval by the ethics committee (Charité Medical Faculty Berlin and University Clinic Erlangen) and written, informed consent, we obtained human tissues. Mesenchymal stromal cells (MSCs) from a non-affected healthy female (23 years) donor were obtained, characterized, and differentiated as previously described [13]. Fibroblasts from buttocks biopsies of a non-affected male donor (25 years) were cultivated until passage six in M-199, supplemented with 20% FCS. Single samples, each from one donor, were used for sequencing.

Patients or patients’ parents signed informed consent on anonymous data collection for research studies conducted on biological samples of patients with primary immunodeficiencies (three Wiskott-Aldrich syndrome samples, four ADA-SCID samples) at San Raffaele Hospital (TIGET02), approved by the San Raffaele Scientific Institute’s Ethical Committee. Four T cell lines were generated from peripheral blood mononuclear cells purified by density gradient centrifugation on Ficoll-Hypaque (Nycomed Pharma, Oslo, Norway) and expanded [14].

### Tissue preparation

Adipose tissue was extracted during lipo-aspiration of MSCs from upper abdominal fat. The tissue was rinsed with PBS on Teflon fleece to wash out erythrocytes. Fat spheres were subsequently frozen in liquid nitrogen. Neutrophils were extracted from peripheral whole blood that was supplemented with 30% of dextran. Cells settled down in a syringe after 30 min. The upper phase was under-laid with histopaque 1083. After centrifugation at 4 °C for 30 min at 1200 rpm, speed was slowed down to 1050 rpm after 15 min for another 15 min. Pelleted cells were resuspended in 10 ml water for water lysis. For neutralization, 3.33 ml of 3.6% NaCl was added for 10 min. After 10 min centrifugation at 1050 rpm, pelleted neutrophils were resuspended in TRIzol® Reagent (Ambion). Plasma, serum, and platelets were prepared using the Vacutainer system. Whole blood in serum tubes was left undisturbed for clot formation. After 15–30 min, the clot was centrifuged at 1000×*g* for 10 min and the supernatant serum was immediately frozen at −80 °C. Plasma was prepared from whole blood in EDTA tubes. After a centrifugation at 2000×*g* for 10 min, the supernatant was frozen. Citrate tubes were used to obtain platelets. The whole blood was centrifuged for 15 min at 100×*g* without rotor break, preventing platelets’ activation. Endothelial progenitor cells (EPCs) were extracted from umbilical cord blood and expanded in vitro using the Lonza EGM™-2 kit. Human umbilical vein cells (HUVECs) were freshly prepared using standard techniques from umbilical cord and cultivated until passage four in EGM medium (Lonza). Adipose tissue, cortex, placenta, decidua, heart, vena cava, muscle, and umbilical cord were minced using pistils and homogenized with matrix beads in the MP FastPrep-24 Tissue and Cell Homogenizer.

### RNA preparation for RNA-seq and qRT-PCR analysis of selected circRNA candidates

RNA was prepared using TRIzol® Reagent (Ambion) and phenol/chloroform precipitation. For Illumina sequencing, rRNA depletion was done with the RiboMinus™ eukaryote kit according to manufacturer’s recommendation (Life Technologies). Bioanalyzer measurement validated the successful rRNA depletion. The Illumina TruSeq sample preparation kit (v2) was used to generate the libraries for sequencing. For qRT-PCR, total RNA of the identical samples that were used for RNA-seq was digested with RNase R (3 U/μg RNA, Epicenter Technologies) and incubated for 15 min at 37 °C with following inactivation for 3 min at 95 °C. To reach similarly effective RNase R treatment, all samples were treated simultaneously in one approach. Then, the RNA was spiked with 10% of *Caenorhabditis elegans* total RNA. After phenol/chloroform precipitation, the RNA was reverse transcribed using RevertAid first strand cDNA synthesis kit (Fermentas) or Maxima RT kit (ThermoFisher Scientific), and SYBR-green quantification (Roche) was performed according to standard protocols on ABI 7500 or StepOnePlus (ThermoScientific). Oligonucleotides flanking the circRNA head-to-tail junctions were designed in Primer3 (v. 0.4.0). RNase R assays were normalized to *C. elegans eif3d* spiked-in RNA and to human *GAPDH* or *Vinculin*. For experiments on WAS and ADA-SCID samples, ΔCt was calculated compared to 28S rRNA. In general, expression was quantified applying the ΔΔCt method. qRT-PCR products were analyzed for amplicon size, specificity, and integrity on 3% agarose gels; concatemers were not taken into account. Sanger sequencing of qRT-PCR products was performed using the Big Dye® Terminator Cycle Sequencing on the 3130xl Genetic Analyzer (ABI) using Gene Mapper® Software Version 4.0. Kit v1.1 (ABI). SeqMan software (Lasergene Version 7.0; DNAStar) was used to analyze the traces.

### circRNA detection and annotation

circRNAs were detected and annotated using the Memczak et al. (2013) pipeline. Human genome reference used for all analyses was hg19 (February 2009, GRCh37), downloaded from UCSC [15]. Upon detection, candidates were annotated using RefSeq and GENCODE v17 gene models. Table S2 and circBase summarize the detected circRNAs across all cell types. Table S2 harbors genomic positions and annotated host transcripts, sense or antisense strand orientation, circBase IDs, genomic and spliced lengths, number of sequencing reads supporting a head-to-tail junction, as well as the number of either 5′ or 3′ linear reads for each circRNA candidate. We also calculated the circular-to-linear ratios and added the list of samples from other studies listed within circBase.

### Data availability

RNA sequencing data have been deposited in the Gene Expression Omnibus (GEO) under the accession number GSE100242.

### circRNA quantification

The ratio of circular and linear isoforms (circular-to-linear ratio, CLR) was calculated as described in [16]. For each circRNA candidate, we counted the number of reads overlapping the head-to-tail junction, and the number of reads spliced linearly over the 5′ and 3′ sites that gave rise to a circRNA. CLR was expressed as the number of reads spanning the head-to-tail junction divided by the number of linear reads mapped over the splice site (5′ or 3′) with the higher read count:

$$ \mathrm{CLR}=\#\mathrm{reads}\_\mathrm{circular}/\max\ \left(\#\mathrm{reads}\_\mathrm{linear}\_5-\mathrm{prime},\#\mathrm{reads}\_\mathrm{linear}\_3-\mathrm{prime}\right) $$

To avoid division by zero when calculating CLR, a pseudocount of 1 was used where no linearly spliced reads were detected. To estimate the expression levels of circRNA host genes, we mapped RNA-seq libraries to the hg19 reference using STAR [17] and counted the reads mapped to Ensembl (release 75) gene models using the htseq-count tool [18, 19].

### circRNA expression heatmaps

**Fig. 1:**
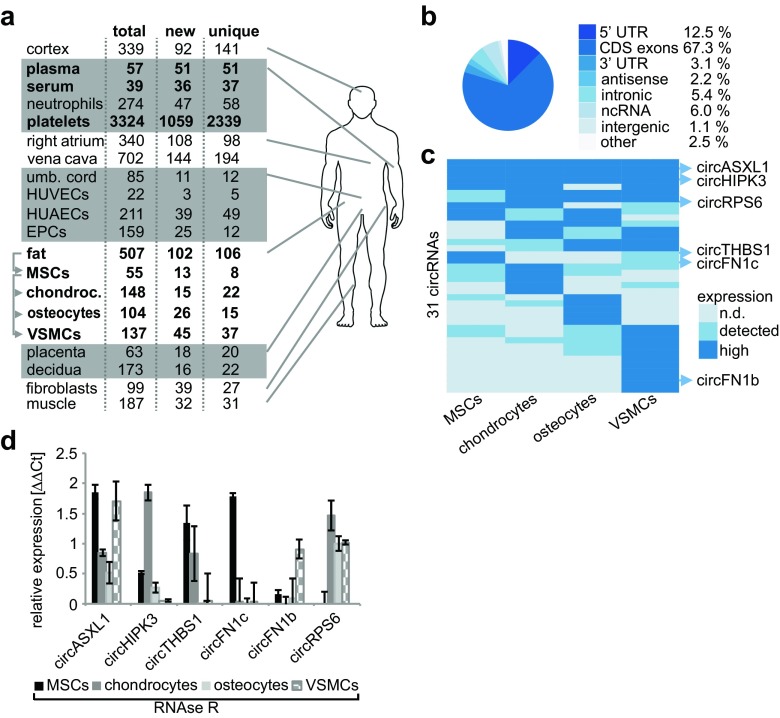
CircRNA expression in clinically relevant human tissues. **a** CircRNA catalog of human samples with total, new, and unique circRNAs. Samples in bold were derived from one donor. **b** Distribution of host gene annotation with potential circRNAs. **c** Differentially expressed top 10% circRNAs in hierarchical clustering. **d** CircRNAs from different clusters (C) were validated by qRT-PCR of RNase R-treated samples

**Fig. 2:**
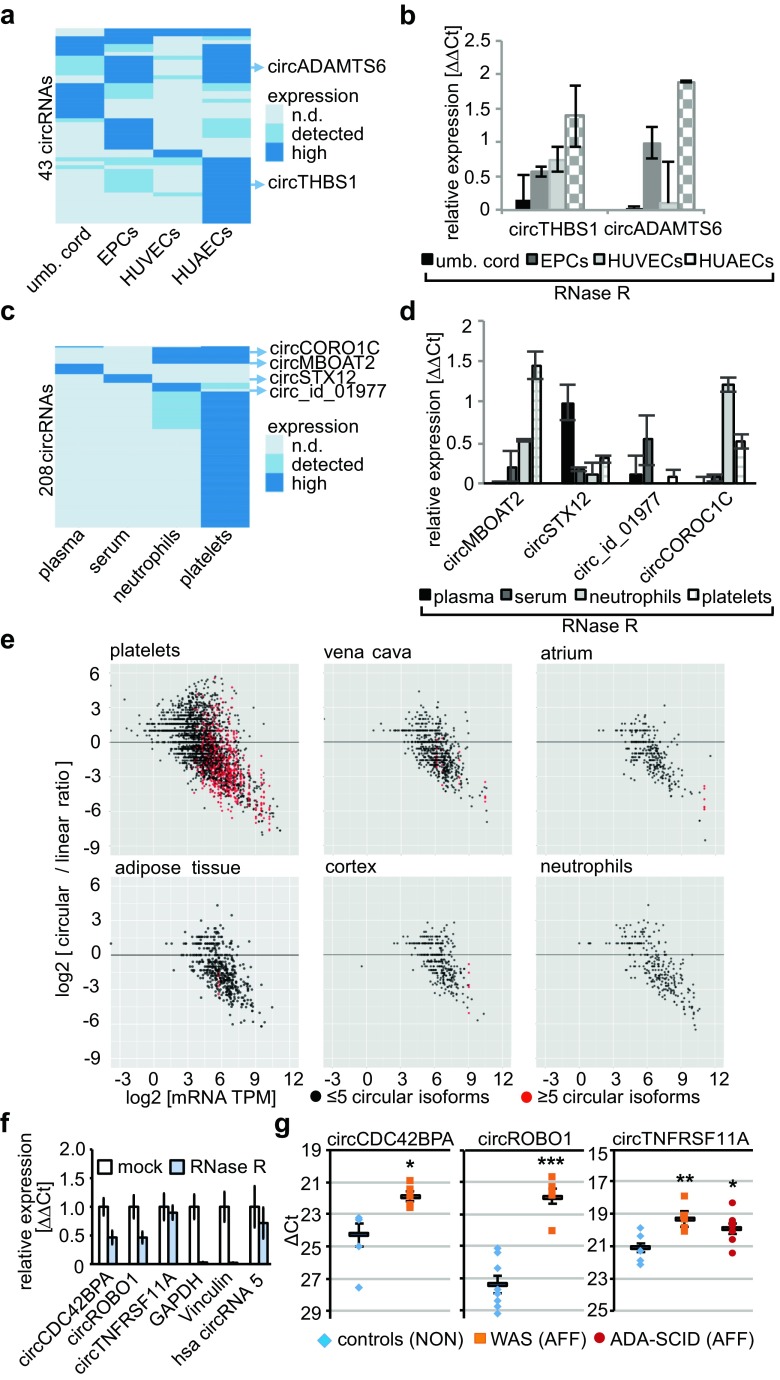
CircRNAs are tissue-specific, highly abundant, and the expression can be differential in disease-associated genes. **a** Clustering of top 10% differentially expressed circRNAs in umbilical cord, EPCs, HUVECs, and HUAECs and **b** their validation. **c** Tissue-specific expression pattern reflected the overlaps of circRNAs in plasma, serum, neutrophils, and platelets and **d** their validation. **e** Circular-to-linear expression ratios revealed highly expressed circRNAs with multiple isoforms. **f** Validation of circRNAs from disease-relevant genes (*GAPDH; Vinculin*—linear negative control; *hsa-circRNA-5*—positive control). **g** CircRNA quantification in three different WAS and four ADA-SCID patients (AFF). The candidates were higher expressed in disease samples than in four controls (bars: mean expression ± SEM, two technical replicates per WAS or ADA-SCID patient group, except *circCDC42BPA*: controls *n* = 6, WAS *n* = 5, Student’s *T* test across samples and replicates, ***p < 0.001, **p < 0.01, *p < 0.05)

circRNAs in Figs. 1c and 2a, c were sorted into three discrete expression classes: (i) “high”—top 10% (5% for platelets) expressed, measured by raw read counts, circRNAs in a particular sample; (ii) “detected”—all circRNAs that satisfied the minimum expression threshold of two unique reads overlapping head-to-tail junction; and (iii) “not detected”—circRNAs that were not detected in a particular sample. Only circRNAs that were assigned to “high” category in at least one of the compared samples were plotted.

### Differential gene expression

Differential gene expression analysis was performed using the DESeq package [20]. method=“blind” and sharingMode=“fit-only” options were used when running the estimateDispersions function, as suggested by the package documentation for experimental designs with no biological replicates.

### In-solution protein digest

Peptides were generated using an automated setup [21]. Briefly, samples were reduced with 1 mM tris(2-carboxyethyl) phosphine (TCEP) and free sulfhydryl groups carbamidomethylated using 5.5 mM chloroacetamide. Proteins were pre-digested with 0.5 μg sequencing grade endopeptidase LysC (Wako) for 3 h at room temperature and subsequently diluted with four volumes of 50 mM ammonium-bicarbonate (ABC). Tryptic digestion occurred for 10 h at room temperature using 1 μg sequencing grade trypsin (Promega). The reaction was stopped by adding trifluoroacetic acid (TFA) to a final concentration of 1% resulting in a final pH of 2. The peptides were purified by using C_18_ stage-tips (3 M) [22].

### Mass spectrometry

Peptides eluted from C_18_ stage-tips were run on an LC-MS setup. The fractionated and unfractionated samples were measured by LC-MS/MS on a Q Exactive orbitrap mass spectrometer (Thermo) connected to a Proxeon nano-LC system (Thermo) in data-dependent acquisition mode using the top 10 peaks for HCD fragmentation. Peptides were separated on an in-house prepared nano-LC column (0.074 mm × 250 mm, 3 μm Reprosil C_18_, Dr. Maisch GmbH). Five microlitres of the sample were injected and the peptides were eluted on a 3-h gradient of 4 to 76% ACN and 0.1% FA in water at flow rates of 0.25 μl/min. MS acquisition was performed at a resolution of 70,000 in the scan range from 300 to 1700 *m*/*z*, MS2 spectra were collected at a mass resolution of 17,000 with a fixed injection time of 120 ms. Dynamic exclusion was set to 30 s and the normalized collision energy was specified to 26. The eluent was directly sprayed into an Q Exactive mass spectrometer (Thermo Fisher Scientific) equipped with a nano electrospray ion source. The recorded spectra were analyzed using MaxQuant software package version 1.5.2.4 [23], with an Andromeda search using the combined UniProt *Homo sapiens* and *Oryctolagus cuniculus* databases and a custom database for the circRNA-derived peptides with a false discovery rate of 1% (peptides and proteins). The fixed and variable modifications were set to carbamidomethylation of cysteines and methionine oxidation, respectively. For further data analysis, the R statistical software package was used (supplement: python script).

## Results

### circRNA resource catalog of 20 human tissues

We generated a circRNA resource catalog for various research interests by sequencing ribosomal RNA-depleted total RNA (Supplemental Table S1). The head-to-tail splice junction identification and the sequence analysis were done according to previously published protocols (Supplemental Fig. S1), [2, 24]. The circRNA catalog can be retrieved in Supplemental Table S2 or circBase, http://www.circbase.org/ [24]. We selected circRNA candidates for validation according to either of the following criteria: (i) the candidate originated from disease or developmental genes (Table 1); (ii) the linear host transcripts have been proposed as biomarkers; (iii) the circRNA was not present in circBase; (iv) the circRNA was encoded from a lncRNA; or (v) the circRNA showed extraordinary genomic length or expression determined via the read count. In total, we selected 112 candidates, of which we validated 71 circRNAs by RNase R assays (validation rate 63.4%, see “Methods”, Supplemental Fig. S2a–p). We normalized expression values to *C. elegans eif3d* spiked-in RNA and to human *GAPDH* or *Vinculin*. Concatamers in putative circRNA candidates were not taken into account.

**Table 1:** Validated circRNAs of known disease-associated genes

circRNA	Disease	Tissue	Circular reads	5′ linear reads	3′ linear reads	CLR	Reference for mRNA
*circPLOD2*	Osteoarthritis-related synovial fibrosis	Osteocytes	2	5	5	0.4	[25]
*circEFEMP1*	Ovarian cancer, glioblastoma	HUVEC	2	29	16	0.1	[26]
*circNTRK2*	Huntington’s disease	Cortex	4	31	96	0	[27–29]
*circRTN4*	Alzheimer’s disease	Cortex	2	104	47	0	[30]
*circHOMER1*	Traumatic neuronal injury, mental retardation, Alzheimer’s disease, schizophrenia, drug addiction	Cortex	9	10	8	0.9	[31, 32]
*circATXN10*	Spinocerebellar ataxia type 10	Cortex	3	18	21	0.1	[33]
*circPSG5*	Pregnancy complications	Placenta	22	26	0	0.8	[34]
*circPAPPA2*	Preeclampsia, fetal growth restriction, HELLP syndrome	Placenta	6	213	172	0	[35–38]
*circALPP*	*Trypanosoma cruzi* infection during pregnancy	Placenta	27	29	0	0.9	[39]
*circNPPA*	Type 2 diabetes, cardiovascular disorders	Heart	9	2695	0	0	[40, 41]
*circCORIN*	Heart failure	Heart, vena cava	2	13	29	0	[42]
*circRYR2*	Atrial fibrillation	Heart, vena cava	2	56	59	0	[43]
*circMYH6*	Hypertrophic cardiomyopathies	Heart	2	364	204	0	[44]
*circSLC8A1*	Cardiovascular disorders	Heart	37	16	51	0.7	[45]
*circPDE3A*	Hypertension	Platelets	5	3	6	0.8	[46, 47]

*CLR* circular-to-linear ratio

Of the 5225 circRNAs, 35.9% (1878 circRNAs) were new compared to circBase [24]. circRNAs (3841) were unique for the investigated cell types (Fig. 1a). We detected 82.9% circRNAs in coding genes (exons, 5′ + 3′ UTRs), 2.2% antisense transcripts, 5.4% intron-derived circRNAs, 6% in non-coding genes, and 1.1% from intergenic regions (Fig. 1b).

### circRNA expression in mesenchymal stem cells and MSC-derived cells

First, we analyzed circRNA expression during MSC differentiation. MSCs were differentiated into proliferating chondrocytes, osteocytes, and vascular smooth muscle cells (VSMCs). Flow cytometry revealed CD105^+^, CD90^+^, CD73^+^, HLA-ABC^+^, CD31^−^, CD34^−^, CD45^−^, and HLA-DR^−^ cells and the multi-lineage potential validated MSC properties [46, 48]. In MSCs, we detected 55 circRNAs, in contrast to 148 in MSC-derived chondrocytes, 104 in osteocytes, or 137 in VSMCs. In chondrocytes, we validated a circRNA deriving from *PLOD2*, a gene which forms collagen crosslinks and was differentially expressed in a model of osteoarthritis-related synovial fibrosis [25]. During the abdominal fat aspiration to obtain MSCs, we additionally harvested adipose tissue that harbors the MSC niches [49]. In the abdominal fat of the same MSC donor, we identified 507 circRNAs. We confirmed a circRNA in *SORBS1*, a gene inhibiting the induction of glucose transport by insulin, and two circRNAs in *PLIN4*, a gene stimulating lipolysis in adipocytes (Supplemental Fig. S2a–d) [50, 51]. When comparing different MSC-derived tissues, we commonly observed different circRNA isoforms spliced from the same host genes in different MSC-derived cells. Solely eight circRNAs overlapped between adipose tissue, MSCs, and their derived cells (Supplemental Table S2). In eight different tissues of one healthy donor, we excluded interindividual differences and found tissue-restricted expression patterns (Fig. 1a). The absence of circRNA housekeeper and direct comparisons of circRNA expressions between different tissues are controversially discussed endeavors. Thus, we selected the top 10% circRNA candidates within MSCs and MSC-derived cells and clustered 31 circRNAs candidates based on their expression levels: “not detected,” “detected” with at least two reads, or “highly expressed” when detected within the top 10% of the candidates (Fig. 1c). We observed ubiquitous expression for some circRNAs and differential expression regarding the MSC-derived cells. We validated selected circRNA candidates within the cluster analysis showing RNase R resistance and confirmed the results of clustering (Fig. 1d and Supplemental Fig. 2q).

### circRNAs in disease-associated genes of clinically relevant tissues

Next, we compared physiologically neighboring tissues: umbilical cord, endothelial progenitor cells (EPCs), HUVECs, and HUAECs. Of the 211 circRNAs in HUAECs, only two overlapped with the other tissues. *EFEMP1*, an angiogenesis promoter, poor prognostic marker in ovarian cancer, and potential therapeutic target in glioblastoma treatment, harbored a circRNA in HUVECs (22 circRNAs) [26, 52]. As previously described, we analyzed the top 10% of the circRNA candidates. A pool of 43 circRNAs showed differential expression and we validated two circRNAs (Fig. 2a, b and Supplemental Fig. 2q).

In cerebral cortex (339 circRNAs), we validated circRNAs spliced from important cerebral genes. *ERC2* is involved in neurotransmitter release and expresses a circRNA [53]. Five circRNA isoforms were found in *ATRNL1*, a gene regulating the energy homeostasis by melanocortins in the hippocampus [54]. Another circRNA is hosted by *NTRK2*, a gene that was associated with synaptic dysfunction in Huntington’s disease and neuronal differentiation and plasticity in hippocampus [27–29]. We validated a circRNA in *RTN4*, a gene inhibiting axonal sprouting and modulating Alzheimer’s disease progression in a mouse model and one of two predicted circRNAs in *HOMER1*, a gene that is involved in synaptic activity and various neurological disorders [30–32]. Three circRNAs were expressed in *ATXN10* that maintains the survival of neurons, studied in the spinocerebellar ataxia type 10 (Supplemental Fig. S2g) [33].

In placenta, we detected 63 circRNAs, in comparison to 173 in decidua; 15 circRNAs overlapped. We confirmed a circRNA in *PSG5*, a gene encoding a pregnancy-specific glycoprotein. Low levels indicate pregnancy complications [34]. Severe early onset preeclampsia, fetal growth restriction, and HELLP syndrome were associated with high expression of *PAPPA2* that encoded two circRNAs [35–38]. *ALPP*, less expressed and active in hyperglycemic and diabetic placentas of pregnant women infected with or without *Trypanosoma cruzi*, harbored a circRNA (Supplemental Fig. S2h) [39].

We also obtained right atrial tissue and vena cava from two children with multiple cardiac defects. Atrium (340 circRNAs) and vena cava (702) had an intersection of 115 circRNAs; 51 overlapped with calf muscle. In atrium, we validated a circRNA in *NPPA*, a gene that was associated with development of type 2 diabetes [40]. *NPPA* is reactivated in response to cardiovascular disorders and converted to its active form by *CORIN*, which harbored four circRNAs (validated in atrium and vena cava) and could be potential biomarkers for heart failure [41, 42]. Atrial fibrillation was linked to the dysfunction of *RYR2*, which also produces seven circRNA isoforms in atrium and ten in vena cava [43]. Mutations in myosin heavy chains cause hypertrophic cardiomyopathies [44]. In myosin *MYH6*, we validated one circRNA. *QKI* is involved in circRNA biogenesis [6], is responsible for cardiovascular development, and encodes two circRNAs in atrium and vena cava [55]. Alterations in the regulation and expression of *SLC8A1* (two circRNAs in atrium) contribute to various cardiovascular symptoms (Supplemental Fig. S2i–k) [45].

### circRNAs and their isoforms in platelets

Platelets expressed 3324 circRNAs. Platelets derive from bone marrow megakaryocytes, lack nuclei and highly abundant mRNA reservoirs, although translational capabilities are intact [56–59]. Previously, high circRNA expression and circRNA properties were described in platelets and this enrichment was associated with transcriptome degradation [60]. We found in our data that platelets harbored much more abundantly expressed circRNAs than any other tissue. For example, circRNA expression in *ACVR2A* and *SMARCA5* was extremely high compared to mRNA (Supplemental Fig. S3). We validated circRNAs in the phosphodiesterases *PDE3A*, *PDE4D*, and *PDE5A*. PDEs hydrolyze cAMP and cGMP to control blood vessel relaxation, cardiac contractility, and inhibition of platelet aggregation [61–64]. *PDE3A* was previously associated with Mendelian hypertension [46, 65]. Moreover, the guanylate cyclase *GUCY1B3* converting GTP into cGMP expressed a circRNA (Supplemental Fig. 2l–o) [66].

CircRNA expression in plasma (57), serum (39), suggested that circRNAs could be secreted, as it was shown earlier for micro- and other RNAs, and indicated by circRNAs identified in cell culture or serum exosomes [67–71].

In neutrophils (274 circRNAs), *TLR6* functions in the innate immune response and harbored a circRNA [72]. Another key component in the immune system expressing a circRNA in neutrophils is *MYO1F*, a class I myosin regulating the host defense against infection [73]. No overlap between plasma, serum, neutrophils, and platelets facilitates the idea of tissue-restricted circRNA expression. For clustering, we used all plasma and serum circRNAs, the top 10% of neutrophils and the top 5% of platelets and validated four candidates (Fig. 2c, d and Supplemental Fig. 2q).

Due to the lack of nuclei and the highly abundant circRNAs in platelets, we hypothesized that circRNAs could serve as templates for translation as recently shown for few circRNA examples in human and fly [74, 75]. Thus, we used RNase R-treated whole platelet RNA to perform in vitro translation experiments followed by highly sensitive mass spectrometry. We derived putative open reading frames (ORF) that span head-to-tail junctions of our circRNA candidates. These predictions were compared to mass-spectrometrically detected peptides. Controls were reticulocyte lysate of the in vitro translation kit, non-RNase R-treated whole platelet RNA, and total protein of the same platelet-donor. Although we detected peptides in the RNase R-treated translated sample and in the cell lysate matching circRNAs in platelets, those candidates did not overlap with head-to-tail junctions (Supplemental Table S3).

We also investigated circRNA isoforms, since we observed around 100 genes hosting more than five different circRNA isoforms. For example, we detected 18 circRNA isoforms derived either from *PTPN12* or *TTN* in platelets, atrium, and vena cava (Supplemental Fig. S4). We compared circRNA expression directly to linear transcript expression, by counting linearly spliced and head-to-tail spliced reads. The number of reads overlapping with the head-to-tail splice junctions was divided by the number of linear splicing events with identical splice sites (Supplemental Table S2). The calculated value was plotted against the transcript copies per million transcripts (TPM) to describe circRNA expression as circular-to-linear ratio (Fig. 2e). Collectively, we detected high circular-to-linear expression ratios in tissues with abundant circRNA expression, e.g., a platelet circRNA in *SMARCA5* had a circular-to-linear ratio of 151:1 (Supplemental Fig. S3b).

### circRNAs are differentially expressed in disease-relevant genes

Finally, we demonstrate differential circRNA expression in ADA-SCID and WAS, two primary immunodeficiencies which are caused by mutations in *ADA* or *WAS*, respectively [76–78]. First, we compared linear transcripts from one patient compared to a non-affected control. We detected significantly differential expression (*p* ≤ 0.05) of 79 mRNAs in ADA-SCID and 19 mRNAs in WAS lymphoblastoid cells (LCLs) (Supplemental Table S4 and Supplemental Fig. S5a, b). The results were consistent with the molecular pathogenesis of both disease phenotypes. For example, upregulated *BANK1* (*p* = 2.30 × 10^−3^, log2-fold change (lfc) = 3.8) or *PBXIP1* (*p* = 3.53 × 10^−2^, lfc = 2.4) mRNAs in ADA-SCID were associated with impaired B cell receptor-induced calcium mobilization or early blocking of B cell development in the bone marrow (Supplemental Table S4) [79, 80].

We next asked whether these differentially expressed linear transcripts harbor also circRNAs with differential expression between patients and controls. We found a circRNA in *ROBO1*, a gene upregulated in ADA-SCID (mRNA: *p* = 9.76 × 10^−6^, lfc = 5.3) and WAS (mRNA: *p* = 3.68 × 10^−4^, lfc = 8.31, Supplemental Table S4). Moreover, *CDC42BPA* expressed an upregulated circRNA in ADA-SCID (mRNA: *p* = 3.46 × 10^−3^, lfc = 4.7) and WAS (mRNA: *p* = 1.93 × 10^−3^, lfc = 7.4). Notably, ROBO1 and CDC42 are linked to the pathogenesis of WAS. Slit-2 and Robo-1 complexes have been described to inhibit the CXCR4/CXCL12-mediated chemotaxis of T cells [81]. Moreover, ROBO1 and ROBO4 bind WAS to induce filopodia formation [82, 83]. Cdc42-dependent WAS activation was also reported [84, 85]. CDC42 is a major regulator of podosome formation and remodels actin during B cell signaling [86, 87], whereas *CDC42BPA* is a downstream effector of CDC42 [88]. B cell signaling is impaired both in ADA-SCID [79, 80] and WAS [89, 90]. In ADA-SCID, we found a circRNA in *TNFRSF11A* (mRNA: *p* = 8.28 × 10^−3^, lfc = 3.3) TNF receptors participate in several pathways altered in ADA-SCID [14]. We first validated the circRNAs in *ROBO1*, *CDC42BPA*, and *TNFRSF11A* (Fig. 2f and Supplemental S6a) and tested next their differential expression in three WAS and four ADA-SCID samples, compared to four non-affected LCL samples (Fig. 2g). circRNA expression of phenotypically relevant genes was higher in the disease samples (Fig. 2g).

## Discussion

Collectively, we provide a circRNA catalog of human tissues relevant to various fields of clinical research. We provide evidence that circRNAs could serve as biomarkers and that circRNA expression profiles could be directly linked to clinically apparent phenotypes. We focused on detecting circRNAs in various single samples. For further analyzing the proposed circRNA candidates as suitable biomarkers, broader studies addressing tissue specificity vs. donor specificity are needed. Our data corroborate recent findings that circRNA expression is highly tissue-specific [2, 16, 91]. We did not find evidence that platelet circRNAs were translated; however, our result does not provide conclusive evidence that circRNAs are not translated, as it highly depends on mass-spec sensitivity. As previously suggested [60], a resistance to RNA degradation can explain the high abundance of circRNAs in platelets. In the absence of transcription, the detected circRNAs could function independently of transcriptional regulation.

As discussed previously [2], circRNAs uncovered in this study could contribute to regulatory networks governing coding gene expression by acting as miRNA target decoys, RNA-binding protein (RBP) sponges, scaffolding molecules, and transcriptional regulators. A circRNA function is further supported by the conserved nature of circRNA expression and the tissue-specific and regulated abundance [16]. Although we can only speculate that currently disclosed circRNAs influence the functions of their linear counterparts, these new isoforms need to be considered when investigating disease-relevant genes. Since circRNA biogenesis can compete with pre-mRNA splicing, this opens up the possibility that the mRNA output from those, oftentimes well studied genes, is controlled by the hitherto unknown circRNA [5].

## Electronic supplementary material

ESM 1 (PY 1 kb).

ESM 2 (PDF 4252 kb).

ESM 3 (XLSX 768 kb).

ESM 4 (XLSX 120 kb).

ESM 5 (XLSX 45 kb).

## References

[1] Jeck WR, Sorrentino JA, Wang K, Slevin MK, Burd CE, Liu J, Marzluff WF, Sharpless NE (2013). Circular RNAs are abundant, conserved, and associated with ALU repeats. RNA.

[2] Memczak S, Jens M, Elefsinioti A, Torti F, Krueger J, Rybak A, Maier L, Mackowiak SD, Gregersen LH, Munschauer M (2013). Circular RNAs are a large class of animal RNAs with regulatory potency. Nature.

[3] Salzman J, Gawad C, Wang PL, Lacayo N, Brown PO (2012). Circular RNAs are the predominant transcript isoform from hundreds of human genes in diverse cell types. PLoS One.

[4] Salzman J, Chen RE, Olsen MN, Wang PL, Brown PO (2013). Cell-type specific features of circular RNA expression. PLoS Genet.

[5] Ashwal-Fluss R, Meyer M, Pamudurti NR, Ivanov A, Bartok O, Hanan M, Evantal N, Memczak S, Rajewsky N, Kadener S (2014). circRNA biogenesis competes with pre-mRNA splicing. Mol Cell.

[6] Conn SJ, Pillman KA, Toubia J, Conn VM, Salmanidis M, Phillips CA, Roslan S, Schreiber AW, Gregory PA, Goodall GJ (2015). The RNA binding protein quaking regulates formation of circRNAs. Cell.

[7] Ivanov A, Memczak S, Wyler E, Torti F, Porath HT, Orejuela MR, Piechotta M, Levanon EY, Landthaler M, Dieterich C (2015). Analysis of intron sequences reveals hallmarks of circular RNA biogenesis in animals. Cell Rep.

[8] Zhang XO, Wang HB, Zhang Y, Lu X, Chen LL, Yang L (2014). Complementary sequence-mediated exon circularization. Cell.

[9] Hansen TB, Jensen TI, Clausen BH, Bramsen JB, Finsen B, Damgaard CK, Kjems J (2013). Natural RNA circles function as efficient microRNA sponges. Nature.

[10] Piwecka M, Glažar P, Hernandez-Miranda LR, Memczak S, Wolf SA, Rybak-Wolf A, Filipchyk A et al (2017) Loss of a Mammalian Circular RNA Locus Causes miRNA Deregulation and Affects Brain Function. Science. 10.1126/science.aam8526 10.1126/science.aam8526 28798046

[11] Suzuki H, Zuo Y, Wang J, Zhang MQ, Malhotra A, Mayeda A (2006). Characterization of RNase R-digested cellular RNA source that consists of lariat and circular RNAs from pre-mRNA splicing. Nucleic Acids Res.

[12] Memczak S, Papavasileiou P, Peters O, Rajewsky N (2015). Identification and characterization of circular RNAs as a new class of putative biomarkers in human blood. PLoS One.

[13] Maass PG, Aydin A, Luft FC, Schachterle C, Weise A, Stricker S, Lindschau C, Vaegler M, Qadri F, Toka HR et al (2015) PDE3A mutations cause autosomal dominant hypertension with brachydactyly. Nat Genet. 10.1038/ng.3302 10.1038/ng.3302 25961942

[14] Cassani B, Mirolo M, Cattaneo F, Benninghoff U, Hershfield M, Carlucci F, Tabucchi A, Bordignon C, Roncarolo MG, Aiuti A (2008). Altered intracellular and extracellular signaling leads to impaired T-cell functions in ADA-SCID patients. Blood.

[15] Rosenbloom KR, Armstrong J, Barber GP, Casper J, Clawson H, Diekhans M, Dreszer TR, Fujita PA, Guruvadoo L, Haeussler M (2015). The UCSC Genome Browser database: 2015 update. Nucleic Acids Res.

[16] Rybak-Wolf A, Stottmeister C, Glazar P, Jens M, Pino N, Giusti S, Hanan M, Behm M, Bartok O, Ashwal-Fluss R (2015). Circular RNAs in the mammalian brain are highly abundant, conserved, and dynamically expressed. Mol Cell.

[17] Dobin A, Davis CA, Schlesinger F, Drenkow J, Zaleski C, Jha S, Batut P, Chaisson M, Gingeras TR (2013). STAR: ultrafast universal RNA-seq aligner. Bioinformatics.

[18] Flicek P, Amode MR, Barrell D, Beal K, Billis K, Brent S, Carvalho-Silva D, Clapham P, Coates G, Fitzgerald S (2014). Ensembl 2014. Nucleic Acids Res.

[19] Anders S, Pyl PT, Huber W (2015). HTSeq—a Python framework to work with high-throughput sequencing data. Bioinformatics.

[20] Anders S, Huber W (2010). Differential expression analysis for sequence count data. Genome Biol.

[21] Kanashova T, Popp O, Orasche J, Karg E, Harndorf H, Stengel B, Sklorz M, Streibel T, Zimmermann R, Dittmar G (2015). Differential proteomic analysis of mouse macrophages exposed to adsorbate-loaded heavy fuel oil derived combustion particles using an automated sample-preparation workflow. Anal Bioanal Chem.

[22] Rappsilber J, Mann M, Ishihama Y (2007). Protocol for micro-purification, enrichment, pre-fractionation and storage of peptides for proteomics using StageTips. Nat Protoc.

[23] Cox J, Mann M (2008). MaxQuant enables high peptide identification rates, individualized p.p.b.-range mass accuracies and proteome-wide protein quantification. Nat Biotechnol.

[24] Glazar P, Papavasileiou P, Rajewsky N (2014). circBase: a database for circular RNAs. RNA.

[25] Remst DF, Blom AB, Vitters EL, Bank RA, van den Berg WB, Blaney Davidson EN, van der Kraan PM (2014). Gene expression analysis of murine and human osteoarthritis synovium reveals elevation of transforming growth factor beta-responsive genes in osteoarthritis-related fibrosis. Arthritis Rheumatol.

[26] Chen J, Wei D, Zhao Y, Liu X, Zhang J (2013). Overexpression of EFEMP1 correlates with tumor progression and poor prognosis in human ovarian carcinoma. PLoS One.

[27] Bramham CR (2008). Local protein synthesis, actin dynamics, and LTP consolidation. Curr Opin Neurobiol.

[28] Plotkin JL, Day M, Peterson JD, Xie Z, Kress GJ, Rafalovich I, Kondapalli J, Gertler TS, Flajolet M, Greengard P (2014). Impaired TrkB receptor signaling underlies corticostriatal dysfunction in Huntington's disease. Neuron.

[29] Yang J, Harte-Hargrove LC, Siao CJ, Marinic T, Clarke R, Ma Q, Jing D, Lafrancois JJ, Bath KG, Mark W (2014). proBDNF negatively regulates neuronal remodeling, synaptic transmission, and synaptic plasticity in hippocampus. Cell Rep.

[30] Masliah E, Xie F, Dayan S, Rockenstein E, Mante M, Adame A, Patrick CM, Chan AF, Zheng B (2010). Genetic deletion of Nogo/Rtn4 ameliorates behavioral and neuropathological outcomes in amyloid precursor protein transgenic mice. Neuroscience.

[31] Fei F, Rao W, Zhang L, Chen BG, Li J, Fei Z, Chen Z (2014). Downregulation of Homer1b/c improves neuronal survival after traumatic neuronal injury. Neuroscience.

[32] Luo P, Li X, Fei Z, Poon W (2012). Scaffold protein Homer 1: implications for neurological diseases. Neurochem Int.

[33] Marz P, Probst A, Lang S, Schwager M, Rose-John S, Otten U, Ozbek S (2004). Ataxin-10, the spinocerebellar ataxia type 10 neurodegenerative disorder protein, is essential for survival of cerebellar neurons. J Biol Chem.

[34] Camolotto S, Racca A, Rena V, Nores R, Patrito LC, Genti-Raimondi S, Panzetta-Dutari GM (2010). Expression and transcriptional regulation of individual pregnancy-specific glycoprotein genes in differentiating trophoblast cells. Placenta.

[35] Buimer M, Keijser R, Jebbink JM, Wehkamp D, van Kampen AH, Boer K, van der Post JA, Ris-Stalpers C (2008). Seven placental transcripts characterize HELLP-syndrome. Placenta.

[36] Macintire K, Tuohey L, Ye L, Palmer K, Gantier M, Tong S, Kaitu'u-Lino TJ (2014). PAPPA2 is increased in severe early onset pre-eclampsia and upregulated with hypoxia. Reprod Fertil Dev.

[37] Varkonyi T, Nagy B, Fule T, Tarca AL, Karaszi K, Schonleber J, Hupuczi P, Mihalik N, Kovalszky I, Rigo J (2011). Microarray profiling reveals that placental transcriptomes of early-onset HELLP syndrome and preeclampsia are similar. Placenta.

[38] Whitehead CL, Walker SP, Ye L, Mendis S, Kaitu'u-Lino TJ, Lappas M, Tong S (2013). Placental specific mRNA in the maternal circulation are globally dysregulated in pregnancies complicated by fetal growth restriction. J Clin Endocrinol Metab.

[39] Mezzano L, Sartori MJ, Lin S, Repossi G, de Fabro SP (2005). Placental alkaline phosphatase (PLAP) study in diabetic human placental villi infected with Trypanosoma cruzi. Placenta.

[40] Jujic A, Nilsson PM, Engstrom G, Hedblad B, Melander O, Magnusson M (2014). Atrial natriuretic peptide and type 2 diabetes development—biomarker and genotype association study. PLoS One.

[41] Houweling AC, van Borren MM, Moorman AF, Christoffels VM (2005). Expression and regulation of the atrial natriuretic factor encoding gene Nppa during development and disease. Cardiovasc Res.

[42] Dong N, Chen S, Wang W, Zhou Y, Wu Q (2012). Corin in clinical laboratory diagnostics. Clin. Chim. Acta.

[43] Dobrev D, Voigt N, Wehrens XH (2011). The ryanodine receptor channel as a molecular motif in atrial fibrillation: pathophysiological and therapeutic implications. Cardiovasc Res.

[44] Jiang J, Wakimoto H, Seidman JG, Seidman CE (2013). Allele-specific silencing of mutant Myh6 transcripts in mice suppresses hypertrophic cardiomyopathy. Science.

[45] Khananshvili D (2013). The SLC8 gene family of sodium-calcium exchangers (NCX)—structure, function, and regulation in health and disease. Mol Asp Med.

[46] Maass PG, Aydin A, Luft FC, Schachterle C, Weise A, Stricker S, Lindschau C, Vaegler M, Qadri F, Toka HR (2015). PDE3A mutations cause autosomal dominant hypertension with brachydactyly. Nat Genet.

[47] Toka O, Tank J, Schachterle C, Aydin A, Maass PG, Elitok S, Bartels-Klein E, Hollfinger I, Lindschau C, Mai K et al (2015) Clinical effects of phosphodiesterase 3A mutations in inherited hypertension with brachydactyly. Hypertension. 10.1161/HYPERTENSIONAHA.115.06000 10.1161/HYPERTENSIONAHA.115.06000 26283042

[48] Salem HK, Thiemermann C (2010). Mesenchymal stromal cells: current understanding and clinical status. Stem Cells.

[49] Vermette M, Trottier V, Menard V, Saint-Pierre L, Roy A, Fradette J (2007). Production of a new tissue-engineered adipose substitute from human adipose-derived stromal cells. Biomaterials.

[50] Baumann CA, Ribon V, Kanzaki M, Thurmond DC, Mora S, Shigematsu S, Bickel PE, Pessin JE, Saltiel AR (2000). CAP defines a second signalling pathway required for insulin-stimulated glucose transport. Nature.

[51] Clifford GM, Londos C, Kraemer FB, Vernon RG, Yeaman SJ (2000). Translocation of hormone-sensitive lipase and perilipin upon lipolytic stimulation of rat adipocytes. J Biol Chem.

[52] Hiddingh L, Tannous BA, Teng J, Tops B, Jeuken J, Hulleman E, Boots-Sprenger SH, Vandertop WP, Noske DP, Kaspers GJ (2014). EFEMP1 induces gamma-secretase/Notch-mediated temozolomide resistance in glioblastoma. Oncotarget.

[53] Takao-Rikitsu E, Mochida S, Inoue E, Deguchi-Tawarada M, Inoue M, Ohtsuka T, Takai Y (2004). Physical and functional interaction of the active zone proteins, CAST, RIM1, and Bassoon, in neurotransmitter release. J Cell Biol.

[54] Haqq AM, Rene P, Kishi T, Khong K, Lee CE, Liu H, Friedman JM, Elmquist JK, Cone RD (2003). Characterization of a novel binding partner of the melanocortin-4 receptor: attractin-like protein. Biochem J.

[55] Justice MJ, Hirschi KK (2010). The role of quaking in mammalian embryonic development. Adv Exp Med Biol.

[56] Bugert P, Dugrillon A, Gunaydin A, Eichler H, Kluter H (2003). Messenger RNA profiling of human platelets by microarray hybridization. Thromb Haemost.

[57] Gnatenko DV, Dunn JJ, McCorkle SR, Weissmann D, Perrotta PL, Bahou WF (2003). Transcript profiling of human platelets using microarray and serial analysis of gene expression. Blood.

[58] Machlus KR, Thon JN, Italiano JE (2014). Interpreting the developmental dance of the megakaryocyte: a review of the cellular and molecular processes mediating platelet formation. Br J Haematol.

[59] Rowley JW, Oler AJ, Tolley ND, Hunter BN, Low EN, Nix DA, Yost CC, Zimmerman GA, Weyrich AS (2011). Genome-wide RNA-seq analysis of human and mouse platelet transcriptomes. Blood.

[60] Alhasan AA, Izuogu OG, Al-Balool HH, Steyn JS, Evans A, Colzani M, Ghevaert C, Mountford JC, Marenah L, Elliott DJ (2016). Circular RNA enrichment in platelets is a signature of transcriptome degradation. Blood.

[61] Beca S, Helli PB, Simpson JA, Zhao D, Farman GP, Jones PP, Tian X, Wilson LS, Ahmad F, Chen SR (2011). Phosphodiesterase 4D regulates baseline sarcoplasmic reticulum Ca2+ release and cardiac contractility, independently of L-type Ca2+ current. Circ Res.

[62] Hunter RW, Mackintosh C, Hers I (2009). Protein kinase C-mediated phosphorylation and activation of PDE3A regulate cAMP levels in human platelets. J Biol Chem.

[63] Mullershausen F, Friebe A, Feil R, Thompson WJ, Hofmann F, Koesling D (2003). Direct activation of PDE5 by cGMP: long-term effects within NO/cGMP signaling. J Cell Biol.

[64] Schwarz UR, Walter U, Eigenthaler M (2001). Taming platelets with cyclic nucleotides. Biochem Pharmacol.

[65] Toka O, Tank J, Schachterle C, Aydin A, Maass PG, Elitok S, Bartels-Klein E, Hollfinger I, Lindschau C, Mai K (2015). Clinical effects of phosphodiesterase 3A mutations in inherited hypertension with brachydactyly. Hypertension.

[66] Zabel U, Weeger M, La M, Schmidt HH (1998). Human soluble guanylate cyclase: functional expression and revised isoenzyme family. Biochem J.

[67] Koh W, Pan W, Gawad C, Fan HC, Kerchner GA, Wyss-Coray T, Blumenfeld YJ, El-Sayed YY, Quake SR (2014). Noninvasive in vivo monitoring of tissue-specific global gene expression in humans. Proc Natl Acad Sci U S A.

[68] Lasda E, Parker R (2016). Circular RNAs co-precipitate with extracellular vesicles: a possible mechanism for circRNA clearance. PLoS One.

[69] Valadi H, Ekstrom K, Bossios A, Sjostrand M, Lee JJ, Lotvall JO (2007). Exosome-mediated transfer of mRNAs and microRNAs is a novel mechanism of genetic exchange between cells. Nat Cell Biol.

[70] Wang K, Zhang S, Weber J, Baxter D, Galas DJ (2010). Export of microRNAs and microRNA-protective protein by mammalian cells. Nucleic Acids Res.

[71] Li Y, Zheng Q, Bao C, Li S, Guo W, Zhao J, Chen D, Gu J, He X, Huang S (2015). Circular RNA is enriched and stable in exosomes: a promising biomarker for cancer diagnosis. Cell Res.

[72] Jang TH, Park HH (2014). Crystal structure of TIR domain of TLR6 reveals novel dimeric interface of TIR-TIR interaction for toll-like receptor signaling pathway. J Mol Biol.

[73] Kim SV, Mehal WZ, Dong X, Heinrich V, Pypaert M, Mellman I, Dembo M, Mooseker MS, Wu D, Flavell RA (2006). Modulation of cell adhesion and motility in the immune system by Myo1f. Science.

[74] Legnini I, Di Timoteo G, Rossi F, Morlando M, Briganti F, Sthandier O, Fatica A, Santini T, Andronache A, Wade M (2017). Circ-ZNF609 is a circular RNA that can be translated and functions in Myogenesis. Mol Cell.

[75] Pamudurti NR, Bartok O, Jens M, Ashwal-Fluss R, Stottmeister C, Ruhe L, Hanan M, Wyler E, Perez-Hernandez D, Ramberger E (2017). Translation of CircRNAs. Mol Cell.

[76] Arredondo-Vega FX, Santisteban I, Daniels S, Toutain S, Hershfield MS (1998). Adenosine deaminase deficiency: genotype-phenotype correlations based on expressed activity of 29 mutant alleles. Am J Hum Genet.

[77] Jin Y, Mazza C, Christie JR, Giliani S, Fiorini M, Mella P, Gandellini F, Stewart DM, Zhu Q, Nelson DL (2004). Mutations of the Wiskott-Aldrich Syndrome Protein (WASP): hotspots, effect on transcription, and translation and phenotype/genotype correlation. Blood.

[78] Sauer AV, Di Lorenzo B, Carriglio N, Aiuti A (2014). Progress in gene therapy for primary immunodeficiencies using lentiviral vectors. Curr Opin Allergy Clin Immunol.

[79] Brigida I, Sauer AV, Ferrua F, Giannelli S, Scaramuzza S, Pistoia V, Castiello MC, Barendregt BH, Cicalese MP, Casiraghi M (2014). B-cell development and functions and therapeutic options in adenosine deaminase-deficient patients. J Allergy Clin Immunol.

[80] Sauer AV, Morbach H, Brigida I, Ng YS, Aiuti A, Meffre E (2012). Defective B cell tolerance in adenosine deaminase deficiency is corrected by gene therapy. J Clin Invest.

[81] Prasad A, Qamri Z, Wu J, Ganju RK (2007). Slit-2/Robo-1 modulates the CXCL12/CXCR4-induced chemotaxis of T cells. J Leukoc Biol.

[82] Prasad A, Kuzontkoski PM, Shrivastava A, Zhu W, Li DY, Groopman JE (2012). Slit2N/Robo1 inhibit HIV-gp120-induced migration and podosome formation in immature dendritic cells by sequestering LSP1 and WASp. PLoS One.

[83] Sheldon H, Andre M, Legg JA, Heal P, Herbert JM, Sainson R, Sharma AS, Kitajewski JK, Heath VL, Bicknell R (2009). Active involvement of Robo1 and Robo4 in filopodia formation and endothelial cell motility mediated via WASP and other actin nucleation-promoting factors. FASEB J..

[84] Cammer M, Gevrey JC, Lorenz M, Dovas A, Condeelis J, Cox D (2009). The mechanism of CSF-1-induced Wiskott-Aldrich syndrome protein activation in vivo: a role for phosphatidylinositol 3-kinase and Cdc42. J Biol Chem.

[85] Takenawa T, Suetsugu S (2007). The WASP-WAVE protein network: connecting the membrane to the cytoskeleton. Nat Rev Mol Cell Biol.

[86] Burbage M, Keppler SJ, Gasparrini F, Martinez-Martin N, Gaya M, Feest C, Domart MC, Brakebusch C, Collinson L, Bruckbauer A (2015). Cdc42 is a key regulator of B cell differentiation and is required for antiviral humoral immunity. J Exp Med.

[87] Kim AS, Kakalis LT, Abdul-Manan N, Liu GA, Rosen MK (2000). Autoinhibition and activation mechanisms of the Wiskott-Aldrich syndrome protein. Nature.

[88] Leung T, Chen XQ, Tan I, Manser E, Lim L (1998). Myotonic dystrophy kinase-related Cdc42-binding kinase acts as a Cdc42 effector in promoting cytoskeletal reorganization. Mol Cell Biol.

[89] Pala F, Morbach H, Castiello MC, Schickel JN, Scaramuzza S, Chamberlain N, Cassani B, Glauzy S, Romberg N, Candotti F (2015). Lentiviral-mediated gene therapy restores B cell tolerance in Wiskott-Aldrich syndrome patients. J Clin Invest.

[90] Castiello MC, Scaramuzza S, Pala F, Ferrua F, Uva P, Brigida I, Sereni L, van der Burg M, Ottaviano G, Albert MH (2015). B-cell reconstitution after lentiviral vector-mediated gene therapy in patients with Wiskott-Aldrich syndrome. J Allergy Clin Immunol.

[91] Szabo L, Morey R, Palpant NJ, Wang PL, Afari N, Jiang C, Parast MM, Murry CE, Laurent LC, Salzman J (2015). Statistically based splicing detection reveals neural enrichment and tissue-specific induction of circular RNA during human fetal development. Genome Biol.

