# Supplementary material for: A map of human circular RNAs in clinically relevant tissues
Source: J Mol Med (Berl). 2017 Aug 25;95(11):1179–89. doi: 10.1007/s00109-017-1582-9 (PMC5660143; doi:10.1007/s00109-017-1582-9)
Supplement: Supplementary file 2 — (PDF 4252 kb). [file 109_2017_1582_MOESM2_ESM.pdf]

Electronic Supplementary Material (ESM)

J Mol Med 2017

Title: A map of human circular RNAs in clinically-relevant tissues

Philipp G. Maass[1, 2, 3, 9*], Petar Glažar[2, 9], Sebastian Memczak[2], Gunnar Dittmar[2], Irene Hollfinger[1, 2], Luisa Schreyer[2], Aisha V. Sauer[4], Okan Toka[5,6], Alessandro Aiuti[4,7], Friedrich C. Luft[1, 2, 8] and Nikolaus Rajewsky[2*]

1 Experimental and Clinical Research Center (ECRC), a joint cooperation between the Charité Medical Faculty and the Max Delbrück Center for Molecular Medicine (MDC), Lindenberger Weg 80, 13125 Berlin, Germany

2 Max Delbrück Center for Molecular Medicine (MDC), Robert-Rössle-Strasse 10, 13125 Berlin, Germany

3 Department of Stem Cell and Regenerative Biology, Harvard University, 7 Divinity Ave, Cambridge, MA 02138, USA

4 Scientific Institute HS Raffaele, San Raffaele Telethon Institute for Gene Therapy (SR-Tiget), 20132 Milan, Italy

5 Children's Hospital, Department of Pediatric Cardiology, Friedrich-Alexander University Erlangen, Loschge Strasse 15, 91054 Erlangen, Germany

6 The German Registry for Congenital Heart Defects, Augustenburger Platz 1, 13353 Berlin, Germany

7 Vita Salute San Raffaele University, Milan, Italy

8 Department of Medicine, Division of Clinical Pharmacology, Vanderbilt University School of Medicine, Nashville, TN, 37235, USA

9 The authors contributed equally to the project

* Corresponding authors:

Philipp Maass: philipp_maass@harvard.edu and Nikolaus Rajewsky: rajewsky@mdc-berlin.de

The authors provide this supplement to give readers additional information about their work.

Table S1. Normalized sequencing statistics for the studied samples. (LCL = Lymphoblastoid Cell Line)

sample	total reads	%rRNA	non-rRNA	%genome	unmapped	circs total	circs QC	norm total	norm QC
ADA-SCID LCL	20.4	31.1	14.1	82.2	2.5	4473	159	99465	53293
adipose	17.9	38.4	11.1	84.3	1.7	2293	507	96986	71272
cartilage	3.4	33.7	2.3	81.9	0.4	1049	0	3747	479
chondrocytes	28.4	80.7	5.5	80.4	1.1	1104	148	67717	43437
cortex	16.4	45.1	9.0	87.2	1.2	2393	343	91765	61470
decidua	19.2	53.1	9.0	86.2	1.2	1989	115	100164	50402
EPC	19.9	54.0	9.2	80.3	1.8	1856	93	99809	58926
fibroblasts	11.3	37.8	7.0	83.0	1.2	2550	69	81844	38675
HUAEC	18.3	34.8	12.0	75.3	3.0	1753	211	103747	83096
HUVEC	13.6	91.2	1.2	75.7	0.3	428	19	29743	7690
LCL	23.0	51.8	11.1	82.7	1.9	3019	204	101740	57733
MSC	14.7	67.6	4.8	79.6	1.0	585	57	65267	42128
muscle	16.7	59.8	6.7	85.6	1.0	1796	109	89450	40168
osteocytes	16.3	65.7	5.6	78.8	1.2	1425	104	78022	55847
placenta	20.5	70.7	6.0	87.4	0.8	667	63	65192	38906
plasma	32.6	1.0	32.3	71.7	9.1	564	57	1109	155
neutrophils	15.9	37.0	10.0	88.2	1.2	1655	274	59767	39193
right atrium	20.7	39.6	12.5	85.2	1.8	2955	227	107733	57484
serum	29.1	2.0	28.5	75.2	7.1	415	39	5548	1725
platelets	24.3	30.1	17.0	86.6	2.3	8843	3324	61364	37316
umbilical cord	18.3	72.7	5.0	79.9	1.0	888	85	58718	31475
vena cava	16.8	26.0	12.5	86.6	1.7	4337	471	103063	52578
VSMC	14.2	44.6	7.9	80.3	1.6	1704	137	79970	56499
WAS LCL	25.1	20.2	20.0	83.3	3.3	5826	1071	123734	90684

Fig. S1.

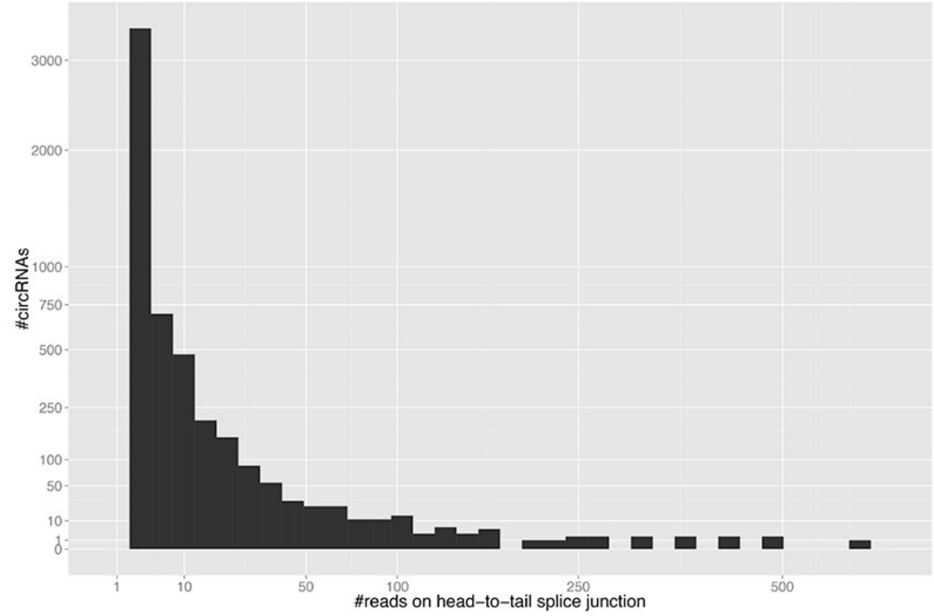

Number of reads on head-to-tail splice junctions of 8760 detected circRNAs.

Fig. S2.

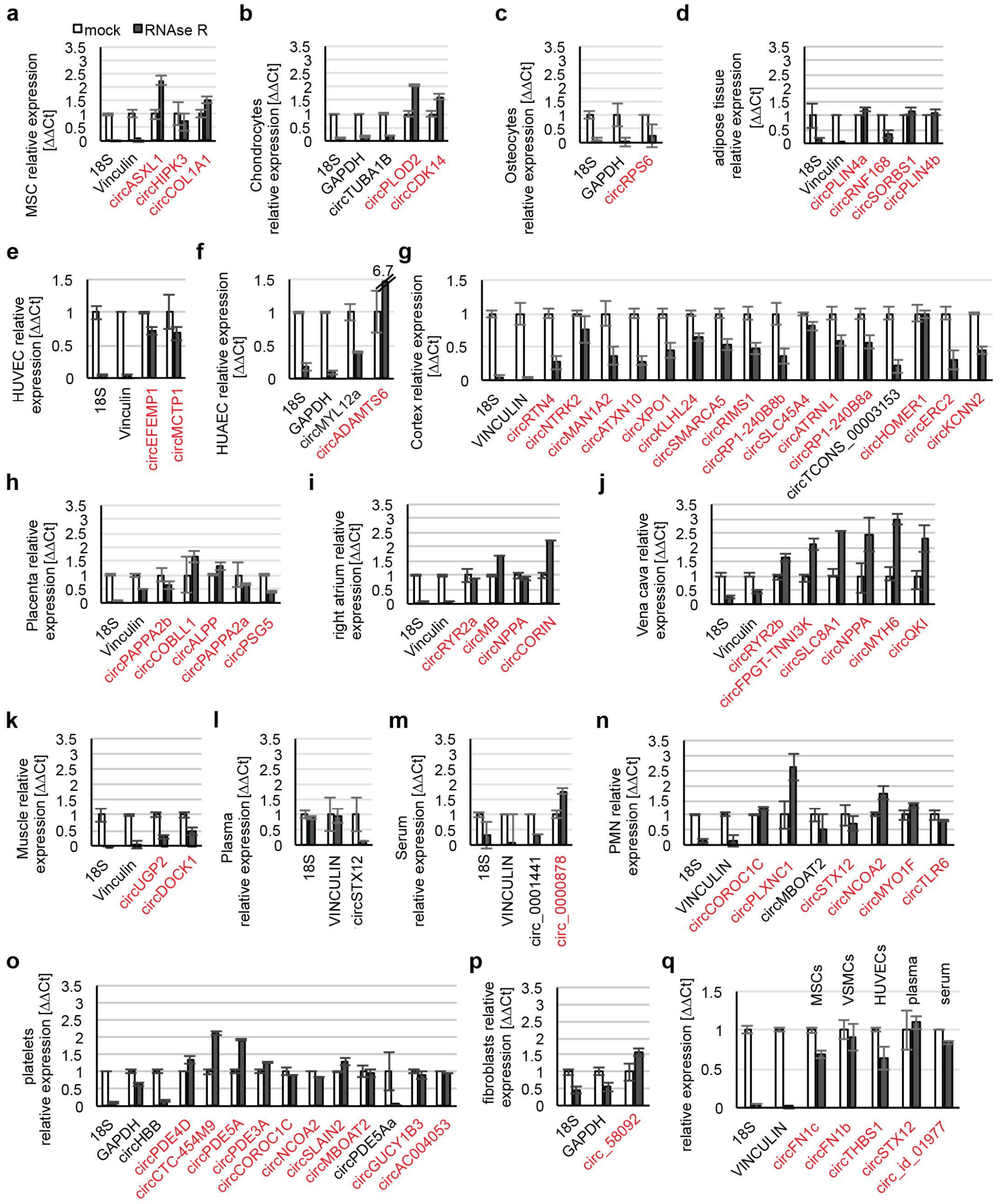

qRT-PCRs validated selected circRNAs (red labels) in various tissues that were at least 5x more resistant to RNAse R treatment.

Fig. S3.

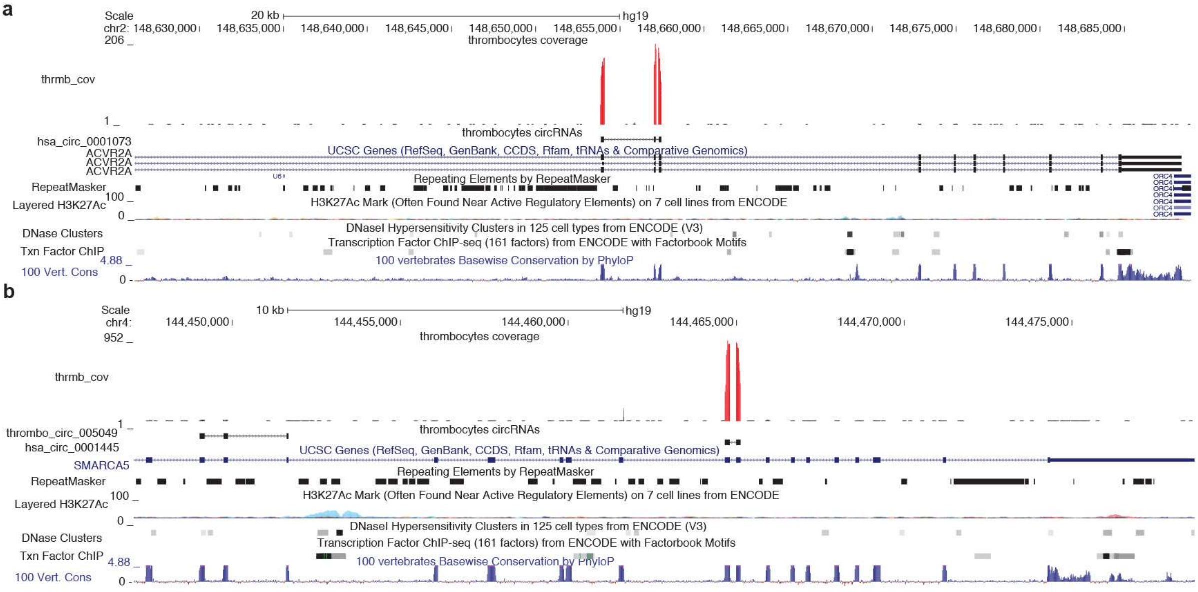

a *ACVR2A* and **b** *SMARCA5* circRNA/mRNA coverage (red peaks) in platelets. The expression of non-circularized exons (black peaks) is barely visible.

Fig. S4.

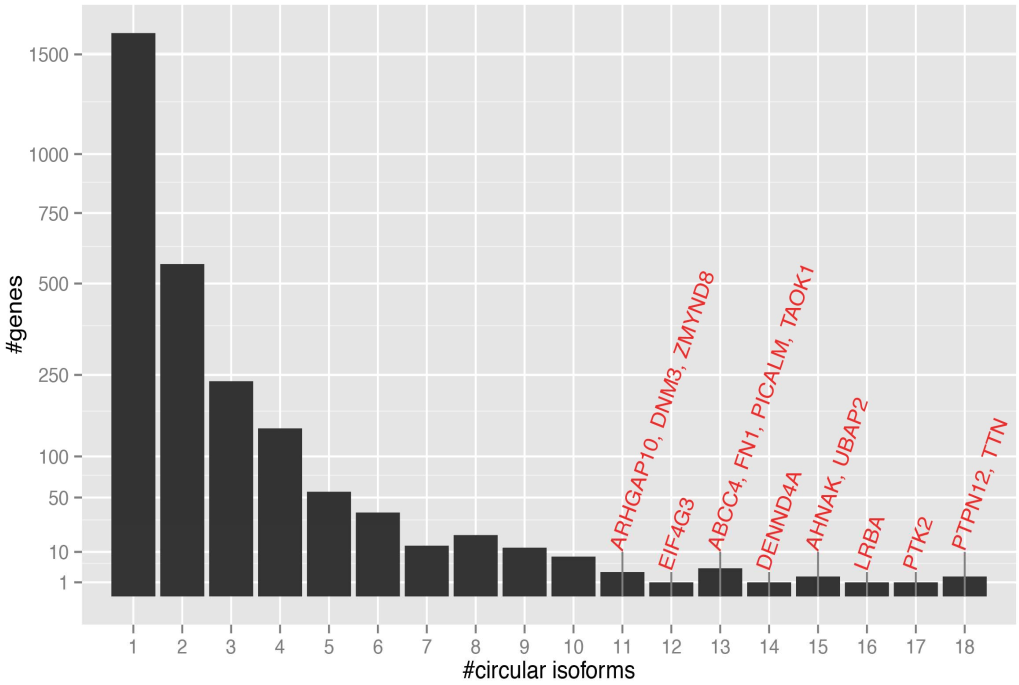

Number of circular isoforms detected in genes hosting circRNAs.

Fig. S5.

a

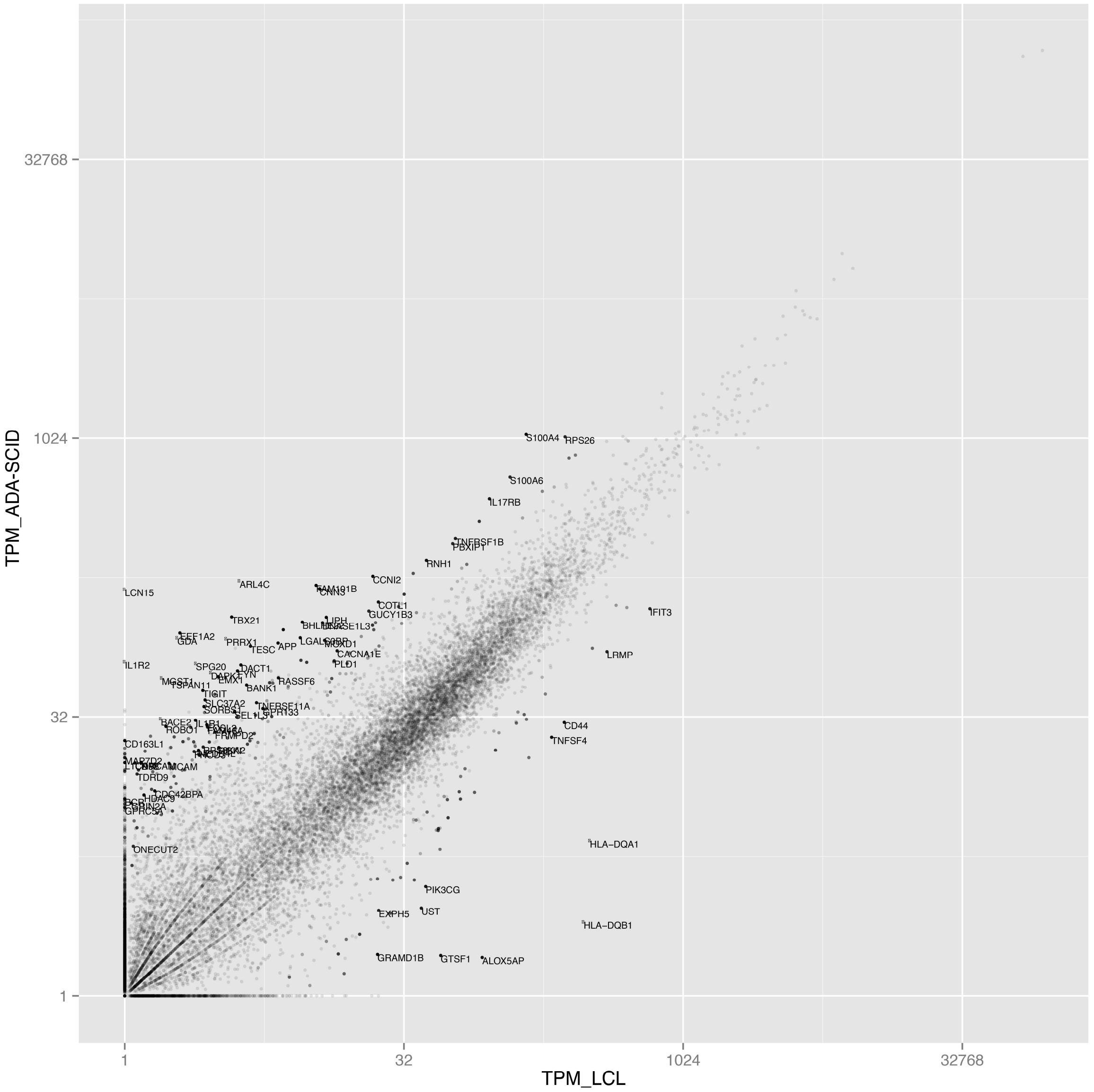
32768
1024
32
1
TPM_ADA-SCID
1
32
1024
32768
TPM_LCL
S100A4
RPS26
S100A6
IL17RB
TNFRSF1B
PBXIP1
RNH1
LCN15
ARL4C
CCNI2
COTL1
GUCY1B3
IFIT3
TBX21
EEF1A2
GDA
PRRX1
TESC
APP
CACNA1E
PLD1
LRMP
IL1R2
SPG20
DACT1
RASSF6
BANK1
MGST1
TSPAN11
TIGIT
SLC37A2
SORBS1
TNFRSF11A
GPR133
CD44
TNFSF4
ROBO1
FRMPD2
CD163L1
TDRD9
CDC42BPA
ONECUT2
HLA−DQA1
PIK3CG
UST
EXPH5
HLA−DQB1
GRAMD1B
GTSF1
ALOX5AP

b

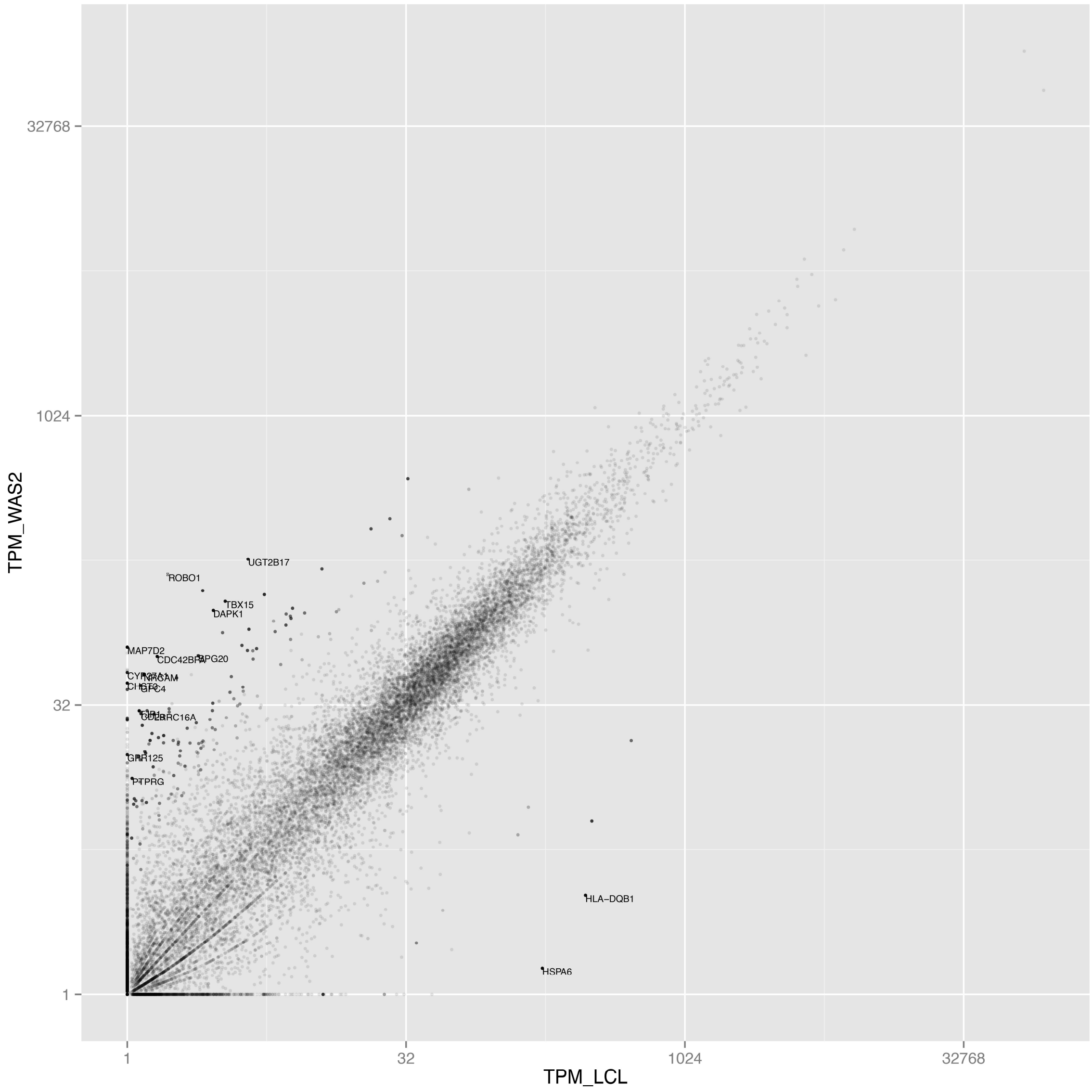

a Differentially expressed linear RNAs in LCLs of one ADA-SCID patient and **b** one patient with Wiskott-Aldrich syndrome (WAS) in comparison to LCLs of a non-affected control.

Fig. S6.

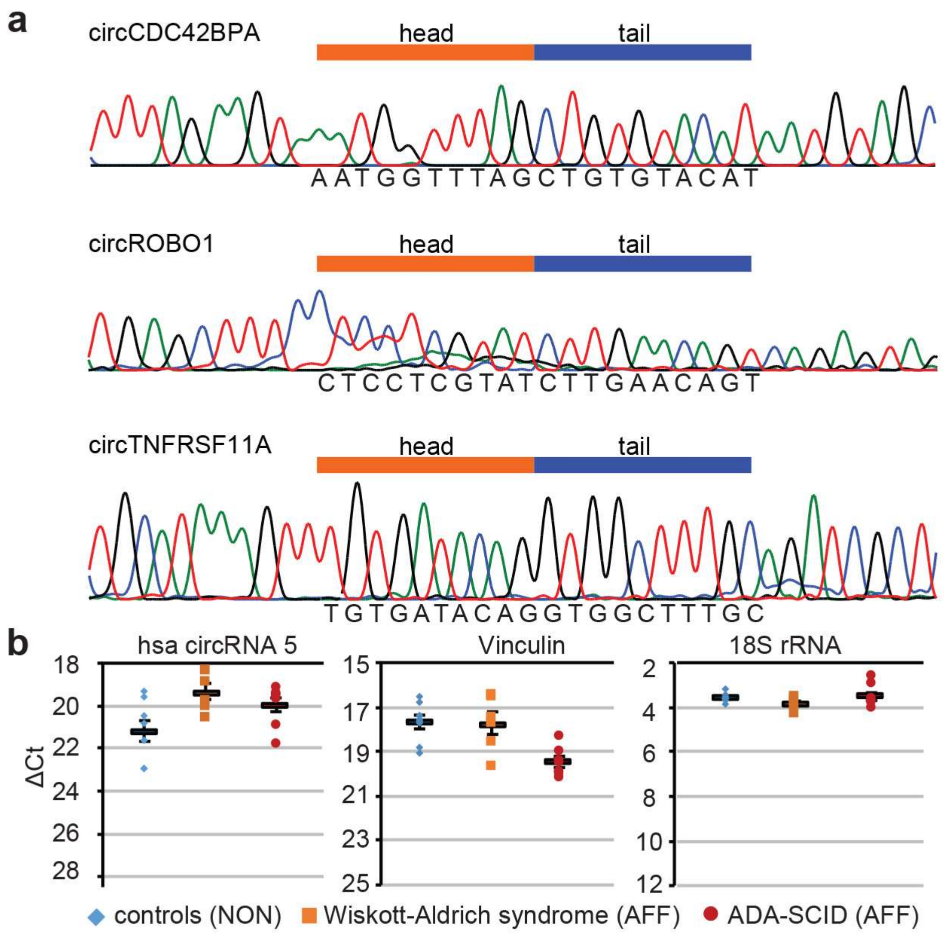

a Electropherograms of Sanger sequencing confirmed head-to-tail splicing of the circRNAs *circCDC42BPA, circROBO1* and *circTNFRS11A*. **b** Controls in qRT-PCR quantifications of 3 Wiskott-Aldrich syndrome samples, 4 ADA-SCID samples, and 4 non-affected LCL controls (technical duplicates per group, bars: mean expression ±SEM, Student's T test, *** $p < 0.001$, ** $p < 0.01$, * $p < 0.05$).
